# Colonic stenting plus vascular plug for malignant gastrocolonic fistula

**DOI:** 10.1055/a-2530-2836

**Published:** 2025-02-17

**Authors:** Kewei Ren, Yahua Li, Zongming Li, Zeyi Yao, Bo Ma, Jianzhuang Ren, Xinwei Han

**Affiliations:** 1 191599 Department of Interventional Radiology, The First Affiliated Hospital of Zhengzhou University, Zhengzhou, China

A 48-year-old man visited our department with a 3-month history of intermediate abdominal pain. In the past 2 months, he had received two cycles of chemotherapy for gastric T-cell lymphoma with colon invasion. Seven days ago, he suffered diarrhea with food debris after a liquid diet. Abdominal computed tomography (CT), gastrointestinal radiography, and gastroscopy revealed the formation of a gastrocolonic fistula and transverse colonic stenosis ( Fig. 1 ). Colonic stenting plus vascular plug placement was developed to solve this problem ( Video 1 ).

**Fig. 1:**
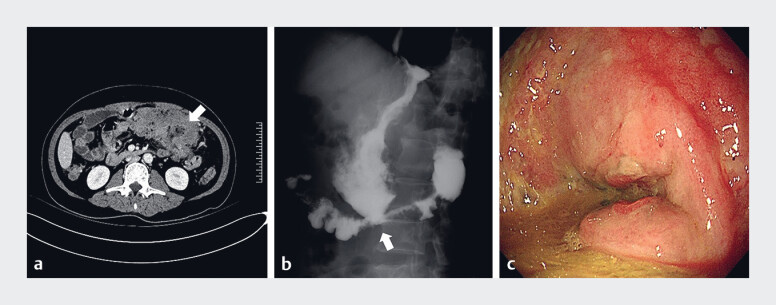
Initial imaging. **a, b** Abdominal computed tomography ( **a** ) and gastrointestinal radiography ( **b** ) revealed formation of a gastrocolonic fistula and transverse colonic stenosis (arrow). **c** The tumor and fistula (arrow) at gastroscopy.

Colonic stenting plus vascular plug for malignant gastrocolonic fistula.Video 1

For this procedure, a 5-Fr curved catheter and guidewire were first introduced to the location of the fistula through the anus. Radiography suggested stenosis of the transverse colon and gastrocolonic fistula formation. A 24 × 120 mm intestinal uncovered stent was successfully placed under fluoroscopic guidance. Then, another 5-Fr vertebral artery catheter and guidewire were introduced through the mouth to the colonic lumen via the gastrocolonic fistula and stent mesh. A 6 × 80 mm balloon was introduced to dilate the stent mesh and insert a 7-Fr sheath. A 16 × 12 mm vascular plug (9-AVP2-016; Abbott, Chicago, Illinois, USA) was introduced. The distal part was released to oppose the stent wall, and the body and proximal part were released in the fistula channel and gastric cavity. Finally, radiography demonstrated that the contrast agent did not enter the colon. Gastroscopy observed the full expansion of the vascular plug in the fistula channel ( Fig. 2 ). CT confirmed the placement of the stent plus vascular plug ( Fig. 3 ). Over the following 2 months, the symptom of diarrhea after eating disappeared. Gastrointestinal radiography did not observe the contrast agent entering the colon.

**Fig. 2:**
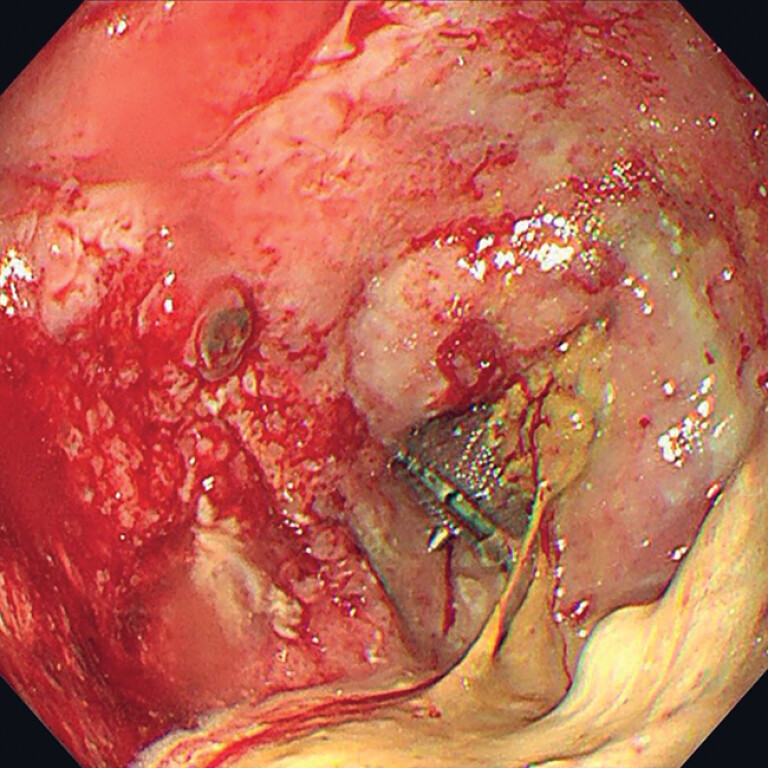
At gastroscopy, full expansion of the vascular plug in the fistula channel was observed.

**Fig. 3:**
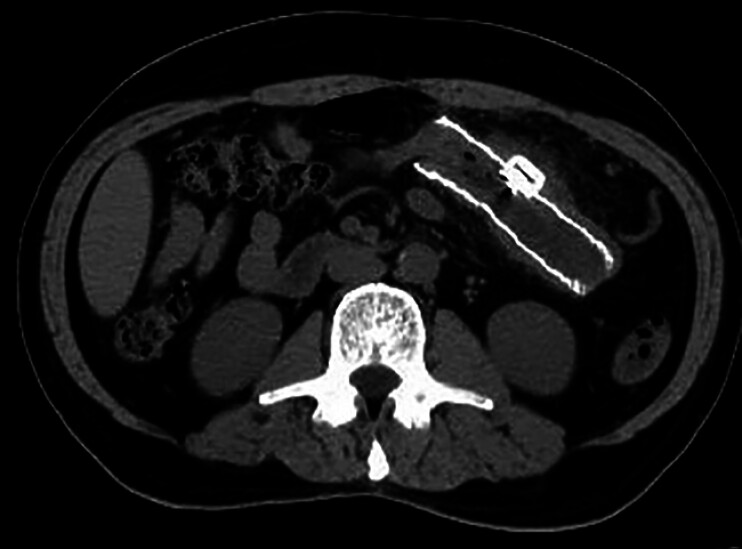
Computed tomography confirmed the placement of the stent plus vascular plug.

The management of gastrocolonic fistula includes surgical repair, opposing stent 1 , and cardiac septal defect closure device 2 . This is the first report of colonic stenting plus vascular plug placement for malignant gastrocolonic fistula, which proved an effective and safe method. Furthermore, the stent acted as an anchor to avoid migration of the vascular plug.

Endoscopy_UCTN_Code_TTT_1AO_2AI

## Notes

Endoscopy E-Videos https://eref.thieme.de/e-videos
*E-Videos* is an open access online section of the journal *Endoscopy* , reporting on interesting cases and new techniques in gastroenterological endoscopy. All papers include a high-quality video and are published with a Creative Commons CC-BY license. Endoscopy E-Videos qualify for HINARI discounts and waivers and eligibility is automatically checked during the submission process. We grant 100% waivers to articles whose corresponding authors are based in Group A countries and 50% waivers to those who are based in Group B countries as classified by Research4Life (see: https://www.research4life.org/access/eligibility/ ). This section has its own submission website at https://mc.manuscriptcentral.com/e-videos .
